# Studies on the Effect of Graphene Oxide Deposited on Gold and Nickel Microsieves on Prostate Cancer Cells DU 145

**DOI:** 10.3390/ijms23126567

**Published:** 2022-06-12

**Authors:** Barbara Nasiłowska, Zdzisław Bogdanowicz, Wiktoria Kasprzycka, Aneta Bombalska, Zygmunt Mierczyk

**Affiliations:** 1 Institute of Optoelectronics, Military University of Technology, gen. S. Kaliskiego 2, 00-908 Warsaw, Poland; wiktoria.kasprzycka@wat.edu.pl (W.K.); aneta.bombalska@wat.edu.pl (A.B.); zygmunt.mierczyk@wat.edu.pl (Z.M.); 2 Faculty of Mechanical Engineering, Military University of Technology, gen. S. Kaliskiego 2, 00-908 Warsaw, Poland; zdzislaw.bogdanowicz@wat.edu.pl

**Keywords:** tumor cells DU 145, microsieves, graphene oxide, sieve column, sieving, Au foil, Ni foil

## Abstract

This work shows the effect of graphene oxide deposition on microsieves’ surfaces of gold and nickel foils, on DU 145 tumor cells of the prostate gland. The sieves were made by a laser ablation process. The graphene oxide (GO) deposition process was characterized by the complete covering of the inner edges of the microholes and the flat surface between the holes with GO. Electron microscanning studies have shown that due to the deposition method applied, graphene oxide flakes line the interior of the microholes, reducing the unevenness of the downstream surfaces during the laser ablation process. The presence of graphene oxide was confirmed by Fourier infrared spectroscopy. During the screening (sieving) process, the microsieves were placed in a sieve column. Gold foil is proven to be a very good material for the screening of cancer cells, but even more so after screening as a substrate for re-culture of the DU 145. This allows a potential recovery of the cells and the development of a targeted therapy. The sieved cells were successfully grown on the microsieves used in the experiment. Graphene oxide remaining on the surface of the nickel sieve has been observed to increase the sieving effect. Although graphene oxide improved separation efficiency by 9.7%, the nickel substrate is not suitable for re-culturing of the Du 145 cells and the development of a targeted therapy compared to the gold one.

## 1. Introduction

Circulating tumor cells (CTC) were first described in 1869 by the Australian physician Thomas R. Ashworth, who rightly stated that the cancer cells in the patient’s blood are very similar or even identical [1]. Cancer cells are excreted from the primary tumor and then they enter the circulatory system as CTCs. A small portion of it may develop into metastatic cancer under appropriate microenvironmental conditions [2,3,4].

A publication by Cristofanilli et al. [5] investigated the CTC concentration in the blood of patients with breast cancer shortly before starting chemotherapy with CellSearch. They observed that patients with a cell count greater than 5 CTC/7.5 mL of blood had less free time to progress than women with less than 5 CTC/7.5 mL of blood [5]. The studies confirmed the prognostic benefit of a CTC assessment in the bloodstream in patients with diagnosed breast cancer. Zhang et al. [6] pointed out that the amount of CTC after chemotherapy indicates a risk of relapse. Circulating tumor cells have also been studied in other solid tumors such as pancreatic cancer, liver cancer, prostate cancer, and melanoma. A correlation between the amount of CTC in the blood with the stage of disease and the outcome of the treatment has been demonstrated [7,8,9,10,11].

In addition, the detection of CTC from the blood of oncologic patients by a so-called liquid biopsy [3] is a non-invasive way for the identification of lesions and may thus form a clinical basis for the development of a targeted therapy [2].

The main problems of CTC detection are the rarity and heterogeneity of their occurrence in the blood, leading to higher costs in the production of adequately effective separators with low interception performance [2]. Therefore, numerous and intensive studies on methods for effective enrichment of CTC [12,13,14,15,16], isolation, and identification [17,18,19,20] have been carried out.

In the process of selecting and detecting the physical properties of circulating tumor cells, the terms size, deformability, density, and electric charge are used [4,21,22,23,24], and for biological properties the terms expression of surface proteins, vitality, and invasiveness. The greatest advantage of physical methods is that cells do not need to be stained and labeled. This increases the ability to cultivate isolated cells and develop targeted therapies. However, the best results of CTC selection and detection have been achieved by combining physical and biological properties [13,15,16].

It has been observed that the use of graphene structures on the surface can increase the vitality of cell cultures. However, due to the different forms, methods of production, origin, and structure of graphene (and its derivatives), the information provided in many publications is quite varied [25]. Some authors point to biocompatibility [25,26], while others report that graphene flakes can sever and damage the cell membrane [27,28,29,30,31]. An undoubtedly interesting research approach was presented in publication [30] in which the results of the study showed that graphene oxide found on the surface of cancer cells cut them off from nutrients.

In addition, the ability to inhibit cell growth has been demonstrated, probably by triggering oxidative stress, which destabilized cell membranes and induced cytoplasm leakage [31]. A significant decrease in the vitality of the graphene oxide-coated cancer cells MDA-MB-231 and SW-954 was observed compared to a cell culture in which a layer of graphene oxide was deposited on the surface of the Petri dish. In the studies presented in [30], it was found that the position of graphene oxide relative to the cells is important. The use of graphene oxide to isolate, capture, identify, and characterize rare CTCs in the blood is a very important issue in the early detection of cancer [32,33,34,35].

In Yoon et al. [32] it is noted that the use of microflow devices is promising, but that these devices are based on three-dimensional structures, which limits the further characterization of circulating tumor cells. Therefore, they proposed isolating CTC from blood samples from patients with a functionalized graphene oxide nano-sheet. This material was used to detect EpCAM proteins on the surface of the CTC [32].

In contrast, Wang and others [34] used reduced graphene oxide (rGO) to isolate circulating tumor cells. The use of reduced graphene oxide resulted in an improvement in the ability to pick up the CTCs from the blood of patients with prostate cancer with an efficacy of up to 60% [34].

Undoubtedly interesting in terms of research and practical approach is the combination of graphene oxide and gold foil to enrich the isolation of circulating new cells [32,36]. Yoon et al. [32] presented the results of a study of a system for the sieving of circulating tumor cells, which also allowed for the storage and multiplication of isolated cells. On a flat silicon substrate, they made gold flower-shaped patterns containing GO nanosheets, and between the patterns was a layer of PDMS (polydimetylosiloxane) forming a microfluidic chamber with a height of 50 μm and a total volume of 45 μL.

Research presented in several papers [4,21,22] has shown that the use of a liquid biopsy in the screening process with the use of microsieves makes it possible to isolate circulating CTC cancer cells.

Although the efficacy of this method was confirmed, no attempt was made to re-cultivate the isolated cells. So far, neither gold nor nickel surfaces have been used as base material, additionally enriched with graphene oxide to separate cancer cells.

Therefore, in order to determine the effect of graphene oxide on cancer cells, screening of DU 145 prostate cancer cells was undertaken on microsieves made of gold and nickel film with an additional embedded coating of graphene oxide.

## 2. Results

### 2.1. Structural Investigations of Au, Au + GO, Ni, and Ni + GO Microsieves

**Figure 1:**
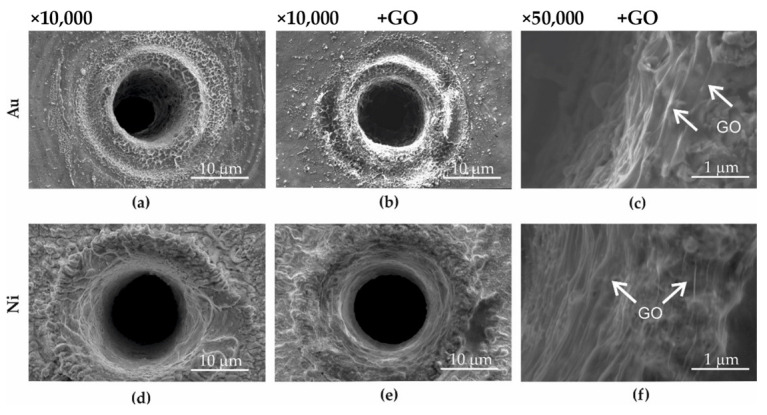
SEM image of the sieves: Au (**a**), Au + GO (**b**,**c**), Ni (**d**) i Ni + GO (**e**,**f**).

Microsieves were made with gold foil—Au (Figure 1a), gold with GO deposited on its surface—Au + GO (Figure 1b,c), nickel foil—Ni (Figure 1d), and nickel with deposited GO—Ni + GO (Figure 1e,f). Procedures for making microsieves by laser ablation, deposition of graphene oxide on the surface of Au + GO and Ni + GO microsieves, and construction of the sieve column are listed in Section 4.1, Section 4.2 and Section 4.3.

As a result of laser ablation (see Section 4.1 on the Au and Ni foil, the local development of the surface underwent significant changes. Between the microholes, the surface has the structure of the base material. On the other hand, in the zone of influence of the laser beam and on the inner edges of the holes, there was an outflow, which during screening could lead to damage to the cell membrane. Therefore, the deposition of graphene oxide on the surface of the microsieves was aimed at reducing the development of the surface and therefore contributing to the separation of the rough surface from the cell membrane.

Studies made by means of a scanning electron microscope (SEM) showed that the deposition of graphene oxide on the surface of the microsieves Au + GO (Figure 1b,c) and Ni + GO (Figure 1e,f) resulted in a GO layer that was located over the entire surface of the sieve. The edges of the graphene sheets overlapped to form a relatively even layer, not only on the flat surface between the holes but also on the top of the microholes and on the inside of the microholes (Figure 1c,f). In addition, it was also observed that the graphene oxide layer reduced the development (roughness) of the surface.

**Figure 2:**
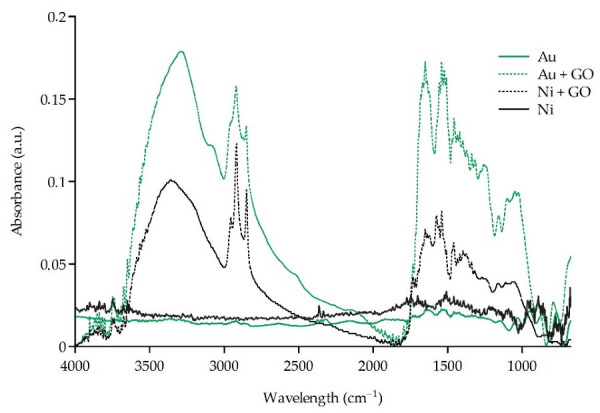
The FTIR spectra of the Au, Au + GO, Ni, and Ni + GO surfaces.

FTIR measurements were performed on the surface of the sieves Au, Au + GO, Ni, and Ni + GO (Figure 2). The FTIR spectra of the Au + GO and Ni + GO sieves were compared to the spectra of microsites without a graphene oxide layer (Au and Ni). The spectral analysis confirmed the presence of a GO layer on the surface of the Au + GO microsieves and the Ni + GO sieves with bonds: –OH in a range of 3358 cm^−1^, C=O in a range of 1650 cm^−1^, C–OH bending in a range of 1540–1575 and 1058 cm^−1^, and non-substituted methyl and methylene groups (CH_3_, CH_2_) were registered at 2849, 2917, 2955 cm^−1^.

Graphene oxide was observed to have a thicker layer on the flat surfaces between the microholes. This may be due to the topography of the microsieves, where the surface of the hole is more developed than the flat surfaces between the holes, but also to the material outflow during laser ablation used for the manufacture of microsites (Section 4.1), which in part acts as a barrier against the dispersed graphene oxide suspension.

### 2.2. Cancer Cell Screening

**Figure 3:**
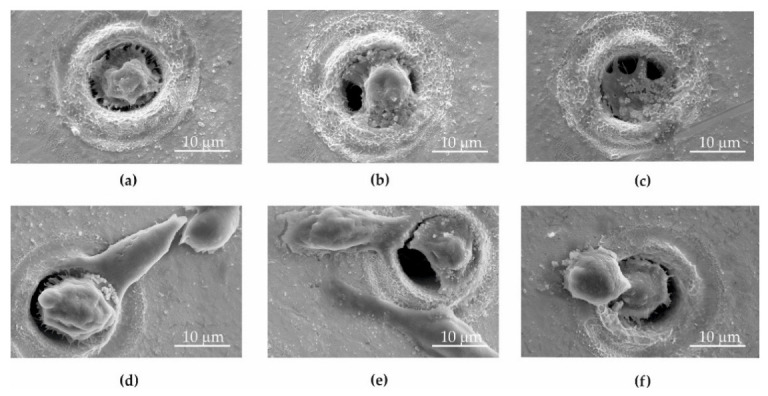
SEM image of the DU 145 cells on Au (**a**–**c**) sieve and Au + GO (**d**–**f**) sieves.

**Figure 4:**
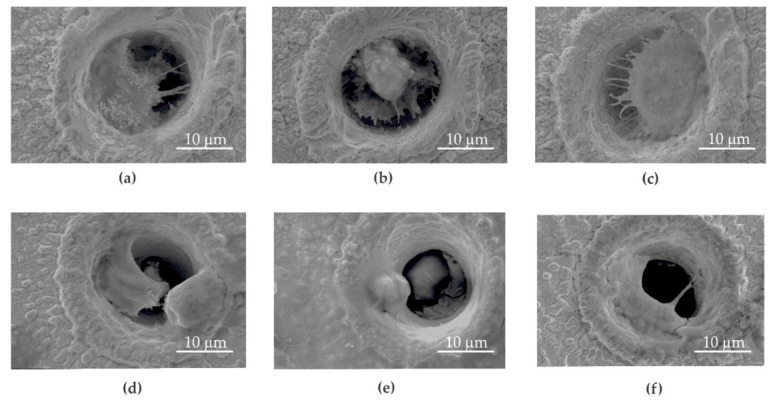
SEM images of a DU 145 cell on the Ni (**a**–**c**) and Ni + GO (**d**–**f**) sieves.

Microsieves made of gold (Au) (Figure 3a), gold and graphene oxide (Au + GO) (Figure 3b,c), nickel (Ni) (Figure 4a), and nickel and graphene oxide (Ni + GO) (Figure 4b,c) were used to separate circulating tumor cells. Procedures to produce microsites, the deposition of graphene oxide and the construction of the sieve column are described in Section 4.1, Section 4.2 and Section 4.3.

Studies on the efficacy of DU 145 screening at the microsieves Au, Au + GO, Ni, and Ni + GO were conducted with the prostate cancer cell line DU 145. A suspension of PBS + DU 145 (10 mL) was placed in a uniform flooding reservoir that leaked through the separator by gravity. During this time, the cancer cells nested in the holes and isolated themselves on the microsieves. The methodology of the cell screening process is described in Section 4.4 and Section 4.5.

**Figure 5:**
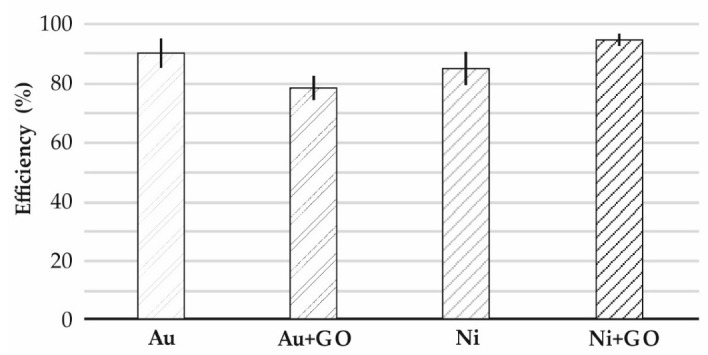
The cell retention efficiency on the microsieves: Au, Au + GO, Ni, Ni + GO.

A high cell retention rate was observed at the microsieves as follows: Au—89.7%, Au + GO—78.4%, Ni—84.8%, and Ni + GO—94.5% (Figure 5).

The microsieve surface analysis conducted by the scanning electron microscope revealed that the sieves made of Au and Au + GO gold foils were far more covered in cancer cells that adhered and proliferated (Figure 3a–f) than the nickel one (Figure 4a–f).

No apoptotic cancer cells were observed on the microsieves made of Au and Au + GO foils. In some places, the number of cancer cells on the gold sieves was significantly higher than in the nickel microsieves. These results coincide with the screening capacity (Au—89.7%, and Ni—84.8%) (Figure 5).

Although the deposition of graphene oxide on the Ni + GO sieves improved the screening quality by around 9.7% compared to the nickel (Ni), the cancer cells did not adhere to the surface and multiply. In addition, the cells nested in the Ni + GO microholes were found to have a different surface morphology. The cell adhesion molecules (CAM) with which the cells adhered to the Ni + GO microsite were very few or none were present. This may have been caused by a graphene oxide layer that has dissolved and deposited on the cells. This situation was not observed with gold microsieves.

### 2.3. Storage and Cell Reculturing

After a study on the screening of DU 145 prostate cancer cells, a new culture was carried out to test the possible development of a targeted therapy.

**Figure 6:**
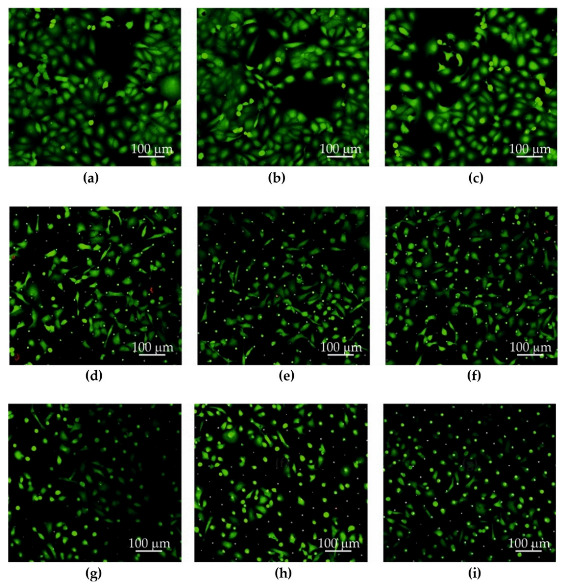
Culture of DU 145 cancer cells on a Petri dish (**a**–**c**), on an Au microsieve (**d**–**f**), and an Au + GO sieve (**g**–**i**). The LIVE/DEAD™ Viability/Cytotoxicity Kit was used for staining (cells stained green—living cells, cells stained red—dead cells).

**Figure 7:**
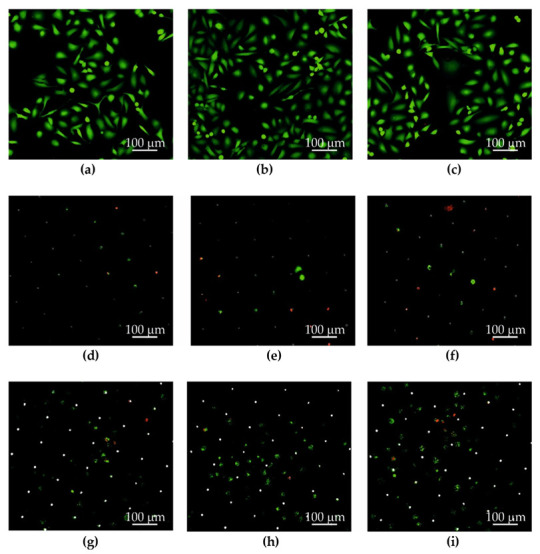
DU 145 cancer cells: culture on a Petri dish (**a**–**c**), on a Ni microsieve (**d**–**f**), and a Ni + GO sieve (**g**–**i**). The LIVE/DEAD™ Viability/Cytotoxicity Kit was used for staining (cells stained green—living cells, cells stained red—dead cells).

The Au, Au + GO, Ni, and Ni + GO microsieves tested were placed in a sterile culture medium (at 37 °C) in the incubator for 24 h after filtration of the PBS + DU 145 (10 mL) suspension. This allowed the adhesion of DU 145 prostate cancer cells to the surface of the separators Au (Figure 6d–f), Au + GO (Figure 6g–i), Ni (Figure 7d–f), Ni + GO (Figure 7g–i), and proliferation. The results of the microsite cell culture studies were compared with a study in which the cells were neither sieved nor cultured through a microsite (Figure 6a–c) and (Figure 7a–c).

For each repetition, the images were taken in different (random) places on a Petri dish. The LIVE/DEAD™ Viability/Cytotoxicity Kit was used for staining (cells stained green—living cells, cells stained red—dead cells) (Figure 6 and Figure 7).

It has been shown that microsieves made of gold foil are best suited for re-breeding. After 24 h, the DU 145 prostate cancer cells were morphologically very similar to those of the control, alive and extended on the surface, indicating good adhesion and ability to continue to cultivate and proliferate.

Although the studies with nickel microsieves showed a high sieving efficiency (Ni—84. 8%), during the new phase of culturing, the cells did not adhere to the surface or proliferate. Only a few nested cancer cells were seen in the microholes (Figure 7d–f). This part of the study showed that pure nickel is unsuitable for further cultivation. It was noted that the Ni + GO microsieves had an increased efficiency of cell screening (94.5%) and the cells were more numerous (Figure 7g–i). However, they were not in good condition as the cells in the control samples (Figure 7g–i). It should also be noted that these cells were not only located in the microholes, but also on the surface of the microsieve. A greater number of dead cancer cells with a damaged cell membrane (red dots) or with metabolism disorder (green patched pattern) were observed, indicating processes that could lead to their death.

## 3. Discussion

In this study, a methodology for the deposition of graphene oxide on microsites from gold and nickel foils by laser ablation was applied. Immediately after RF plasma purification, dispersed graphene oxide was applied to the microsieves, and then alternately centrifugal force and compressed air were applied to the surface of the microsite (parallel to the axis of the openings). The samples were placed in a vacuum dryer to evaporate excess water from the GO suspension.

The idea of the plasma interaction with the surface examined was to improve the hydrophilicity [37,38] of the dispersed aqueous suspension containing graphene oxide flakes and to allow the suspension to flow freely in the microholes despite roughness and surface development.

The method for isolating cancer cells differed from the methods described in publications [16,17], which used mainly systems (chips) made of partially or fully transparent polymer materials. Graphene and graphene derivatives are rarely used, although studies have shown that the screening efficiency of circulating tumor cells has been improved [33,34,35,36]. The main purpose of these devices is to separate and diagnose cells. However, the proposed solution in this trial also provides a re-culturing process that can be used to develop a targeted therapy.

The studies showed that DU-145 prostate cancer cells were successfully separated on microsieves made of gold foil. In order to reduce the production costs of the separators, the tests were carried out not only on gold but also on nickel microsieves.

Deposition efficiency on nickel microsieves (Ni) was quite high at 84.8%, but deposition of graphene oxide (Ni + GO) increased the deposition efficiency by 9.7%.

After the preparation of the cells (Section 4.8), scanning electron microscopy showed that cancer cells on the gold microsieves adhered to the surface and multiplied in large numbers. In some microholes, there was more than one nested cancer cell. In addition, the shape of these cells indicates their well-being and their biocompatibility with gold. Although the cells were treated with formalin (Section 4.8) which was used in the SEM preparation, microcell adhesion molecules were visible in SEM images.

In addition, the cancer cells adhered very well to the gold microsieves during the new culture and proliferated in a good manner. In the SEM images, only individual cells were observed. A definite, large proportion of DU145 cancer cells were characterized by well-being and did not deviate from the control cells (Figure 6a–c) being in very good condition (Figure 6g–i).

It should be noted that the cells were exposed to several negative factors during and after the screening, such as centrifugation, exposure to temperature fluctuations, leaching from the medium and other enveloping media (such as antibiotics), and mechanical action on the cell membrane upon contact with the micro sieve. However, these factors did not adversely affect their proliferation on the gold microsites.

Although the screening effectiveness of D145 tumor cells for nickel microscreens was also high, these cells detached from the surface of the microsite during culture. An improvement in quality of about 9.7% was observed for microsieves with deposited graphene oxide.

The cells which were deposited on a nickel microsieve with a graphene oxide layer showed a different surface topography. A negligible number of microfibres with which cancer cells usually attach themselves to the surface were observed. Probably the graphene oxide flakes detached from the surface and remained attached to the cell membrane, which favored their death. Although the same method of deposition of graphene oxide was used for all types of Au + GO and Ni + GO microsieves, this phenomenon was not observed on gold foil microsites. In addition, no membrane damage was observed during tumor cell sieving.

## 4. Materials and Methods

The samples for the study of the effect of graphene oxide deposited on the microsieves were determined as follows:

- Au—gold microsieve (99.95%), produced by laser ablation (Section 4.1)
- Au + GO—gold microsieve with deposited GO (99.95%), produced by laser ablation (Section 4.1) + graphene oxide deposition process (Section 4.2)
- Ni—nickel microsieve (99.9%) produced by laser ablation (Section 4.1)
- Ni + GO—nickel microsieve with GO deposited on its surface (99.9%) produced by laser ablation (Section 4.1) + graphene oxide deposition (Section 4.2)

### 4.1. Microsieve Manufacture by Laser Ablation

Gold and nickel foils were used to produce the microsieves, which were purchased by Goodfellow GmbH (Goodfellow, Hamburg, Germany) with the dimensions 100 mm × 100 mm × 0.025 mm.

All microsieves used were manufactured by laser ablation using the PL2210/SH/TH/FH picosecond laser from EKSPLA (EKSPLA, Vilnius, Lithuania). A diagram of the laser system with a complete description is presented in publication [39]. The impulse energy was controlled with the polarization silencer 990-0070-355 manufactured by EKSPLA (EKSPLA, Vilnius, Lithuania) [39].

A galvanometric scanner manufactured by the company RAYLASE (RAYLASE, Weßling, Ger-many) with a telecentric length equalization of 100 mm, with a focus on the radiation, was used on the surface of the processed material. The parameters to produce Au, Au + GO, Ni, and Ni + GO microsieves are given in Table 1.

### 4.2. GO Deposition

The microsieves labeled with Au + GO and Ni + GO after the laser ablation process were covered with graphene oxide. This process was carried out using a dispersed aqueous suspension prepared by the Hummers process [30] with a concentration of 4.5 g/L (Department of Chemical Synthesis and Flake Graphene, Łukasiewicz Research Network, Institute of Electronic Materials Technology, Warsaw, Poland). After laser ablation, the microsieves (Au + GO and Ni + GO) were cleaned with an RF plasma (prep III device, Garfield Ave, Westchester, PA, USA). The following RF plasma cleaning parameters were used: 3 h and 100 W. Immediately after removal from the plasma chamber, a dispersed aqueous suspension was applied and exposed to an airflow directed perpendicular to the surface of the sample (parallel to the hole axis) (1 bar, 60 s) (Stanley, Smart Marketing Group Pty Ltd., Mt Waverley, VIC, Australia). In a further step, the samples were subjected to a centrifugal force (1500 rpm, 60 s) on spin coating (Polos, Midden Engweg 41, 3882 TS Putten, The Netherlands), followed by an airflow directed perpendicular to the test surface (parallel to the hole axis).

In order to sterilize the surface of the microsieve with deposited GO oxide and at the same time minimize the effects on the graphene oxide structure, the microsieves were first subjected to a temperature of 120 °C for 600 s in the vacuum dryer Vacucell 22 L (BMT Medical Technology s. r. o., Brno-Zábrdovice, Czech Republic) and UV lamp coupled with a laminar chamber (HERA Safe KS18, Thermo Scientific Langenselbold, Germany) for 1800 s. Structural investigations (including scanning electron microscopy—SEM and Fourier infrared spectroscopy—FTIR) of graphene oxide deposited on microsites were performed after the entire deposition and sterilization process.

### 4.3. The Construction of a Screening Column

**Figure 8:**
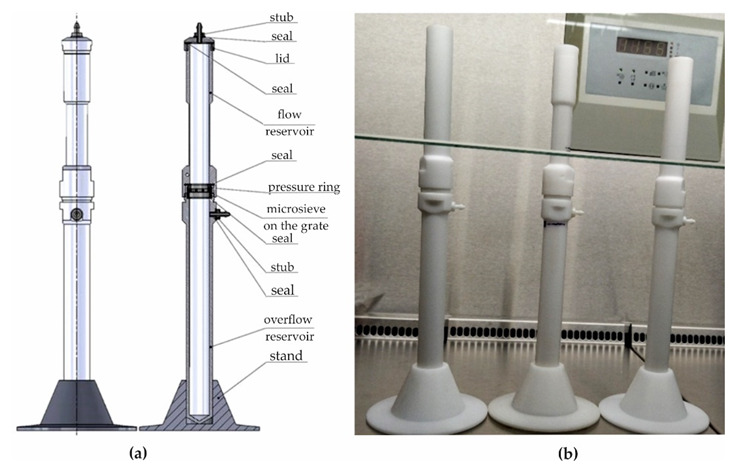
Scheme (**a**) and photo (**b**) of the screening column.

The screening column designed (Figure 8a) and constructed (Figure 8b) allows the isolation of cancer cells in the blood analysis of cancer patients during screening (Military University of Technology, Warsaw, Poland). The main components of the sieve column were a flood and overflow reservoir and a separator that included a microsieve, which was used depending on the intended test: Au, Au + GO, Ni, or Ni + GO. The microsieve was placed on the grate and pressed with a ring (Military University of Technology, Warsaw, Poland).

In addition, the screen carrier column was equipped with a hand pressure pump, a blood pressure meter, and a sealing kit. During the tests, however, the separation was carried out by gravity without any additional pressure change. In the upper container, there was a calibrated hole for positioning the temperature sensor. The threaded connection between the lower part of the standard flood tank and the upper part of the drop tank made quick replacement of the separators coupled to the microsieves possible, as well as easy operation and maintenance of the entire sieve column.

Cell separation of the cancer cells was performed under sterile conditions. All elements of the separation column were subjected to a temperature of 121 °C for 25 min in an autoclave Tuttnauer EL-D (Tuttnauer, 25 Power Dr, Hauppauge, NY 11788, USA).

### 4.4. Cell Culture

The studies were performed with an epithelial morphology cell line isolated from a DU 145 tumor prostate cell (ATCC HTB-81, Manassas, VA, USA). The breeding was done in Eagle’s Minimum Essential Medium, Gluta-MAX, (Gibco^®^, Grand Island, NY, USA) with 10% bovine serum (Gibco^®^, Grand Island, NY, USA) and 1% Antibiotic-Antimycotic (Gibco^®^, Grand Island, NY, USA) at 37 °C and 5% CO_2_ in the air.

The culture was carried out to approx. 80% confluence, then the cells were detached from the substrate using a solution of 0.25% Trypsin-EDTA, (Invitrogen^TM^, Waltham, MA, USA). After the cells were stained with 4% Trypan Blue (Invitrogen^TM^, Waltham, MA, USA) and their density measured in suspension using an automatic Countess II cell reader (Invitrogen, Waltham, MA, USA), they were centrifuged at 130 rcf for 600 s and diluted to 10^4^ cells/mL with PBS (Gibco^®^, Grand Island, NY, USA). The cellular suspension consisted of 92–96% living cells, depending on the repetition sample. The mean size of the living tumor cells was ~22 μm.

### 4.5. Filtration Process

A suspension of PBS + DU 145 (10 mL) was administered using a serological pipette under sterile conditions, which leaked through gravity through a separator containing a microsite (Au, Au + GO, Ni, or Ni + GO) (Military University of Technology, Warsaw, Poland) suitable for the experiment. The number of cells per sieve was 10^4^ cells/mL. Subsequently, the microsieves containing the separated cells were placed in a sterile culture medium (at 37 °C) in an incubator for 24 h (HERA Cell VIOS 160i, Thermo Scientific, Langenselbold, Germany).

The filtrate deposited on microsites was collected. The cells were concentrated to 60 µL using a centrifuge (130 rcf, 600 s) (Centrifuge 5810R, Ependorf, Hamburg, Germany). In the next step, the number of cells in the filtrate was checked using the Countess II automatic reader, Invitrogen^TM^.

For all considered cases of DU 145 cell screening on microsieves (Au, Au + GO, Ni, or Ni + GO), ≥3 tests were performed. The results of the tests together with the standard deviation are presented in Figure 5.

### 4.6. Viability Studies after Filtration

In order to test the feasibility of developing targeted therapy on separated cells, they were re-evaluated in culture. To make the cells adhere to the surface of the microsites after the filtering, they were placed in a sterile culture medium and left for 24 h at 37 °C in the incubator. After this time, the medium was removed and replaced with a solution of LIVE/DEAD™ Viability/Cytotoxicity Kit, Invitrogen (Waltham, MA, USA) dyes consisting of calcein-AM and homodimer ethidium-1 in PBS (Gibco^®^, Grand Island, NY, USA).

Calcein-AM stains the cells with normal metabolism (intracellular esterase activity) green, whereas ethyidine-1 homodimer does not enter the cell until the cell membrane is broken and then binds to nucleic acids to stain them red. The staining protocol was used according to the manufacturer’s instructions (Invitrogen, Waltham, MA, USA). The uniformly green cells were in a good shape after the dye was applied, and the red oval objects were remnants of dead cells (their stained cell nuclei). The cells with the green mosaic pattern and the red center are cells with a disturbed metabolism and a damaged cell membrane.

### 4.7. Microsieves Structural Studies

#### 4.7.1. Fourier-Transform Infrared Spectroscopy—FTIR

The surfaces of all the sieves were examined by using a Thermo Scientific Nicolet iN10 (ThermoFisher SCIENTIFIC, Waltham, MA, USA). The spectra were recorded using the FTIR technique in reflection mode with a nitrogen-cooled MCT detector. The spectra were recorded with 8 cm^−1^ resolution, 128 scans in a range of 4000–675 cm^−1^. Each presented spectrum has an average of 10 different scanned spots.

#### 4.7.2. Scanning Electron Microscope—SEM

All the types of microsieves, Au, Au + GO, Ni, and Ni + GO were characterized using scanning electron microscopy (SEM) (Quanta 250 FEG SEM, FEI, Hillsboro, OR, USA). The SEM image was created with a distributed detector (ETD-BSE, FEI, Hillsboro, OR, USA) with an acceleration voltage of 5 kV for GO and 10 kV.

### 4.8. Imaging of Cancer Cells

#### 4.8.1. Scanning Confocal Microscope

Images of stained cells on microsieves were imaged using a scanning confocal microscope LSM 700 Axio Observer.Z1, Zeiss (Carl Zeiss Microscopy, Jena, Germany).

#### 4.8.2. Scanning Electron Microscope—SEM

The cells on the microsieves were rinsed twice with PBS at 37 °C after 24 h cultivation (an analogous process to that with the LIVE/DEADTM Viability/Cytotoxicity Kit). They were then fixed for 30 min at room temperature in a mixture of 4% formaldehyde and 0.4% glutaraldehyde and PBS physiological saline solution. After preservation, the reagent residues were removed by rinsing the micro sieves with the cells twice with demineralized water.

Due to the lack of cell conductivity, the samples were dried in a Vacucell 22 L vacuum dryer (BMT Medical Technology s.r.o., Brno-Zábrdovice, Czech Republic) and then a 5.12 nm gold layer was deposited on their surface using an EM ACE 600 high-vacuum sputterer (Leica Microsystems, Wetzlar, Germany). To ensure an even spray, the table was rotated at an angle of 120° during the process.

Studies of cancer cell lines separated on Au, Au + GO, Ni and Ni + GO microsieves were performed using scanning electro-new microscopy (SEM) (Quanta 250 FEG SEM, FEI, Hillsboro, OR, USA).

The SEM image was obtained using a backscattered detector (ETD-BSE, FEI, Hillsboro, OR, USA) with an accelerating voltage of 5 kV for GO and 10 kV for cancer cells (all variants).

## 5. Conclusions

Studies of the effect of graphene oxide deposited on microsieves made of gold and nickel film on DU 145 prostate cancer cells showed high screening efficiency reaching Au—89.7%, Au + GO—78.4%, Ni—84.8%, and Ni + GO—94.5%, respectively.

As a result of the process of deposition of graphene oxide on microsieves, the presence of graphene flakes was observed on the entire surface of the microsites, and adjacent to the walls of microholes.

Microsieves after separating the cells were subjected to a 24-h culture process, during which it was observed that the best material for the production of microsieves is gold. Using scanning confocal microscopy, numerous clusters of cells were observed that were in very good condition.

The deposition of graphene oxide had a positive effect on the nickel foil by increasing the effectiveness of cell screening by 9.7%. Although a very good material for insulation, nickel microsieves are not suitable for cell culture. The cells probably peeled off from the substrate during preparation. This indicates that the recovery of cancer cells is better performed on microsieves made of nickel foil with an embedded layer of graphene oxide or gold, while re-culture is better performed on microsieves made of the gold film.

## 6. Patents

Patents resulting from the work reported in this manuscript: PL 223,437 B1, 2016—Molecular Sieve and PL 70,912 Y1, 2019—Separation Columns, Utility design.

## Figures and Tables

**Table 1:** Parameters used in microsieve production.

Ablation Parameters	Au and Au + GO	Ni and Ni + GO
Laser impulse energy	100–110 (µJ)	100–110 (µJ)
Number of impulses of the laser beam	25 (-)	25 (-)
Laser beam radiation—first harmonic	1.3 (mJ)	
wavelength—first harmonic	1064 (nm)	
Laser beam radiation—third harmonic	0.45 (mJ)	
wavelength—third harmonic	355 nm	
Laser beam radiation—fourth harmonic	0.25 (mJ)	
wavelength—fourth harmonic	266 nm	
Laser impulse time	70 (ps)	
Repetition	1 (kHz)	
Microsieve diameter	ϕ23 mm	
Microholes diameter	10–12 (µm)	
Microholes offset	50 (µm)	
No of microholes	~10^5^ (-)	

## Data Availability

Not applicable.
